# Evaluation of the collagen-binding properties and virulence of killed *Streptococcus mutans* in a silkworm model

**DOI:** 10.1038/s41598-022-06345-x

**Published:** 2022-02-18

**Authors:** Yuto Suehiro, Ryota Nomura, Saaya Matayoshi, Masatoshi Otsugu, Naoki Iwashita, Kazuhiko Nakano

**Affiliations:** 1 Department of Pediatric Dentistry, Osaka University Graduate School of Dentistry, 1-8 Yamada-oka, Suita, Osaka 565-0871 Japan; 2 Laboratory of Veterinary Pharmacology, Azabu University, 1-17-71 Fuchinobe, Chuo-ku, Sagamihara, Kanagawa 252-5201 Japan

**Keywords:** Microbiology, Bacteriology

## Abstract

*Streptococcus mutans*, a major pathogen of dental caries, is also known as a causative agent of cardiovascular disease. A 120 kDa collagen-binding protein (Cnm) of *S. mutans* is an important contributor to the pathogenicity of cardiovascular disease. Although dead bacteria have been detected in cardiovascular specimens by molecular biological methods, the pathogenicity of the bacteria remains unknown. Here, we analyzed the pathogenicity of killed *S. mutans* by focusing on collagen-binding ability and the effects on silkworms. In live *S. mutans*, Cnm-positive *S. mutans* had high collagen-binding activity, while Cnm-negative *S. mutans* had no such activity. After treatment with killed Cnm-positive *S. mutans*, amoxicillin-treated bacteria still had collagen-binding ability, while lysozyme-treated bacteria lost this ability. When live and amoxicillin-treated *S. mutans* strains were administered to silkworms, the survival rates of the silkworms were reduced; this reduction was more pronounced in Cnm-positive *S. mutans* infection than in Cnm-negative *S. mutans* infection. However, the administration of any of the lysozyme-treated bacteria did not reduce the survival rate of the silkworms. These results suggest that amoxicillin-killed Cnm-positive *S. mutans* strains maintain collagen-binding properties and pathogenicity in the silkworm model, and are possibly associated with pathogenicity in cardiovascular diseases.

## Introduction

The oral cavity contains not only live bacteria, but also bacteria that have been killed by the administration of antibiotics or by antimicrobial substances in saliva^1,2^. Live and dead bacteria present in the oral cavity can infiltrate the bloodstream when bleeding occurs following invasive dental treatment or daily tooth brushing^1^. Such bacterial invasion into the blood can induce cardiovascular diseases such as infective endocarditis (IE) or arteriosclerosis when bacterial adhesion occurs because of bacterial infections on the vascular walls, especially under abnormal conditions^3,4^. In Gram-positive bacteria, the adhesion properties of live bacteria are considered to be an important risk factor for IE^5–7^, while the pathogenicity of dead bacteria is not well understood.

*Streptococcus mutans*, a major pathogen of dental caries, is also known as a causative agent of IE^8^. Cnm, a 120 kDa collagen-binding protein, is expressed on the cell surface of *S. mutans* at a frequency of approximately 10–20% in bacteria isolated from the oral cavity^9,10^. Cnm-positive *S. mutans* strains can adhere to the vascular wall by virtue of their collagen-binding properties^11^, which are closely associated with the pathogenicity of IE. It has also recently been reported that Cnm-positive *S. mutans* strains are frequently isolated from the oral cavity of patients with some cerebrovascular diseases, such as cerebral microbleeds and intracerebral hemorrhage^12,13^.

A blood culture method is widely used for culturing live bacteria that cause IE^4^. Recently, molecular biological methods that can detect the bacterial DNA of live bacteria as well as dead bacteria have been developed^4^. Previous studies reported that bacterial DNA of *S. mutans* was frequently detected in extirpated heart valve specimens from patients with IE using molecular biological methods, even when no live *S. mutans* was isolated from the patients by the blood culture method^14^. Additionally, genes encoding collagen-binding proteins were frequently detected in these *S. mutans*-positive heart valve specimens^15^. These results led us to hypothesize that dead *S. mutans* may be a possible virulence factor for cardiovascular diseases. In the present study, we analyzed the pathogenicity of Cnm-positive and Cnm-negative *S. mutans* strains killed by amoxicillin, a major antibiotic used for the prevention of IE, and lysozyme, an antimicrobial substance in saliva and serum, using a collagen-binding assay and a silkworm model.

## Results

### Morphological evaluation of live *S. mutans* and killed *S. mutans* (treated with amoxicillin or lysozyme)

**Figure 1:**
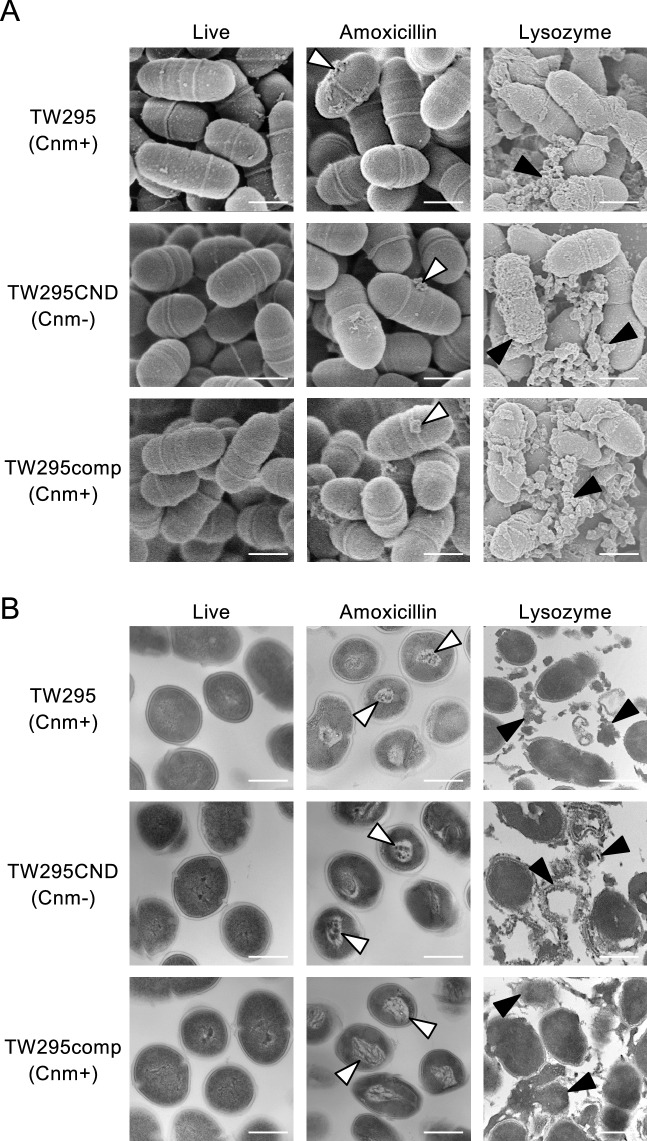
Representative electron microscopy images of live *S. mutans*, amoxicillin-killed *S. mutans*, and lysozyme-killed *S. mutans*. (**A**) Scanning electron microscopy images. Scale bar, 500nM. White and black arrowheads indicate damage to the bacterial cell surface layer and lysis of the bacteria, respectively. (**B**) Transmission electron microscopy images. Scale bar, 500 μm. White and black arrowheads indicate abnormal changes in the cytoplasm and lysis of the bacteria, respectively.

We used *S. mutans* strain TW295, a Cnm-positive strain isolated from individuals with bacteremia after tooth extraction, TW295CND, a Cnm-defective isogenic mutant strain of TW295, and TW295comp, a Cnm-complemented mutant strain of TW295. The killed *S. mutans* strains were prepared by treatment with either amoxicillin or lysozyme. Scanning electron microscopy (SEM) images showed slight damage in the cell surface layer of *S. mutans* treated with amoxicillin (Fig. 1A), and transmission electron microscopy (TEM) images showed abnormal changes in the cytoplasm of the bacteria (Fig. 1B). After the lysozyme treatment, *S. mutans* was lysed in SEM and TEM images (Fig. 1A,B).

### Collagen-binding activity of live *S. mutans*, and killed *S. mutans* (treated with amoxicillin or lysozyme)

**Figure 2:**
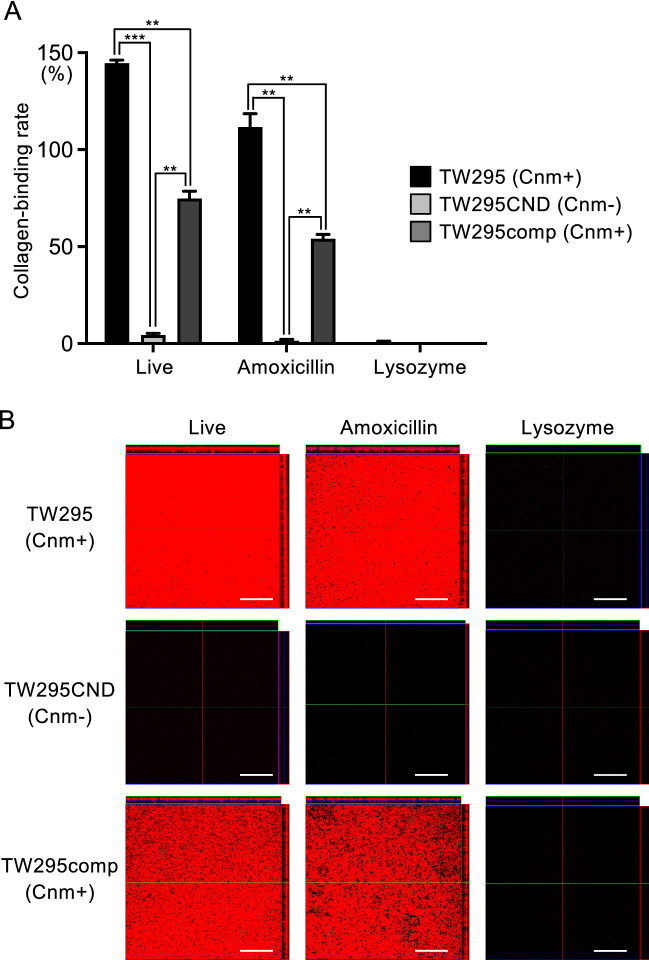
Collagen-binding properties of live *S. mutans*, amoxicillin-killed *S. mutans*, and lysozyme-killed *S. mutans*. (**A**) Collagen-binding rates of *S. mutans* strains. Significant differences were observed using analysis of variance with Bonferroni correction (**P < 0.01 and ***P < 0.001). (**B**) Representative confocal laser scanning microscopy images of *S. mutans* binding to collagen. Bacterial cells are stained red. Scale bar, 50 μm.

In live *S. mutans*, TW295 displayed high collagen-binding activity. This binding activity was dependent on Cnm expression, as shown by the lack of binding by TW295CND, and the recovered binding demonstrated by TW295comp (Fig. 2A,B). After amoxicillin treatment, collagen-binding ability was also observed in the Cnm-positive strains, although the collagen-binding ability was lower than that of live bacteria. In contrast, the collagen-binding ability of all these bacteria was lost following lysozyme treatment.

### Silkworm larvae virulence assay with different doses of live *S. mutans*

**Figure 3:**
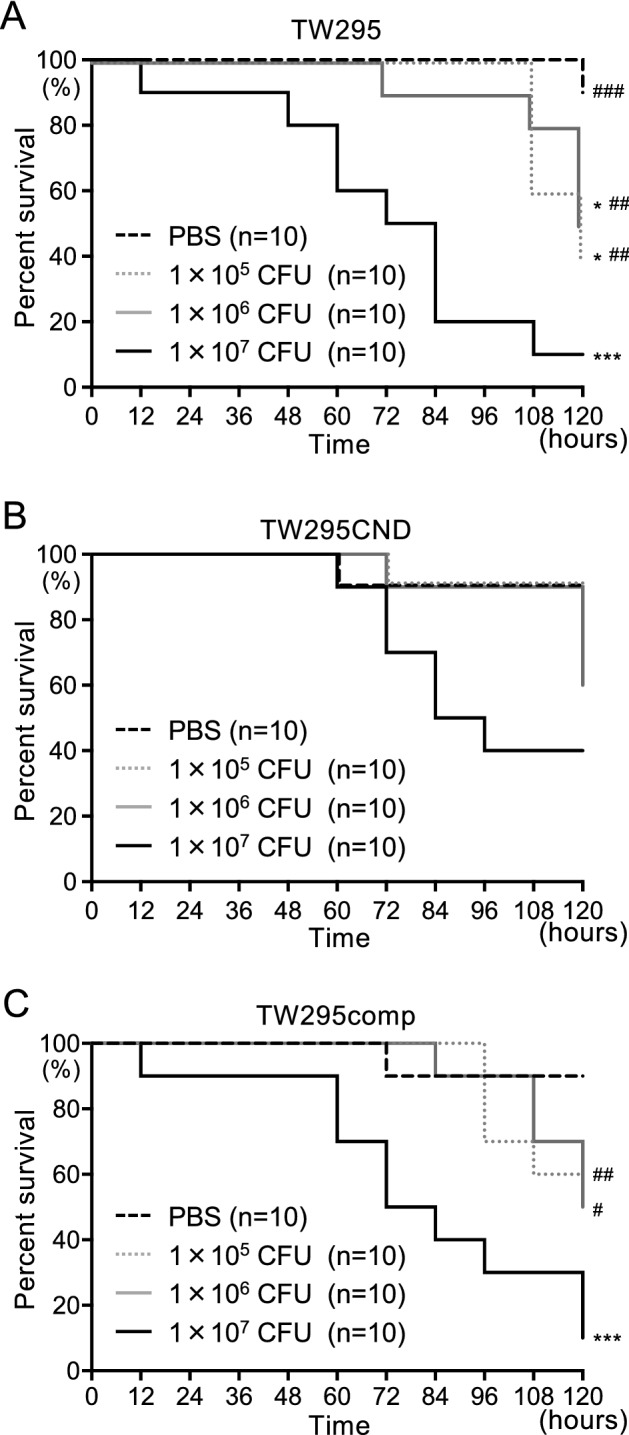
Survival curve for silkworm larvae infected with different doses of live *S. mutans*. Silkworm larvae infected with (**A**) TW295 (Cnm +), (**B**) TW295CND (Cnm−), and (**C**) TW295comp (Cnm +). Survival rates in each group were evaluated in a Kaplan–Meier plot, which was analyzed by a log-rank test. ^*^P < 0.05 and ^***^P < 0.001 versus phosphate-buffered saline group; ^#^*P* < 0.05, ^##^*P* < 0.01, and ^###^*P* < 0.001 versus 1 × 10^7^ CFU of *S. mutans* administration group.

Silkworm (*Bombyx mori*) larvae have similar susceptibility to bacterial infections as humans, and have been used to assess the pathogenicity of bacteria^16^. As a quantitative analysis, different amounts of live *S. mutans* (1 × 10^5^ colony-forming units [CFU], 1 × 10^6^ CFU, and 1 × 10^7^ CFU) were administered to the silkworms. When 1 × 10^5^ CFU and 1 × 10^6^ CFU of TW295 were administered, silkworm mortality commenced late in the experimental period, reaching 50% at 120 h (Fig. 3A). When 1 × 10^7^ CFU of TW295 was administered, silkworm mortality commenced at 12 h after the start of the experiment and reached 50% within 72 h. When 1 × 10^5^ CFU and 1 × 10^6^ CFU of the TW295CND was administered, 50% mortality has not been reached at the end of the experimental period (Fig. 3B). When 1 × 10^7^ CFU of TW295CND was administered, 50% mortality was reached 84 h after the start of the experiment, which was later than that of TW295 for the same dose of bacteria. TW295comp administration resulted in a similar survival curve of mortality as TW295 administration for each dose of bacteria (Fig. 3C).

### Silkworm larvae virulence assay of live *S. mutans* and killed *S. mutans* (treated with amoxicillin or lysozyme)

**Figure 4:**
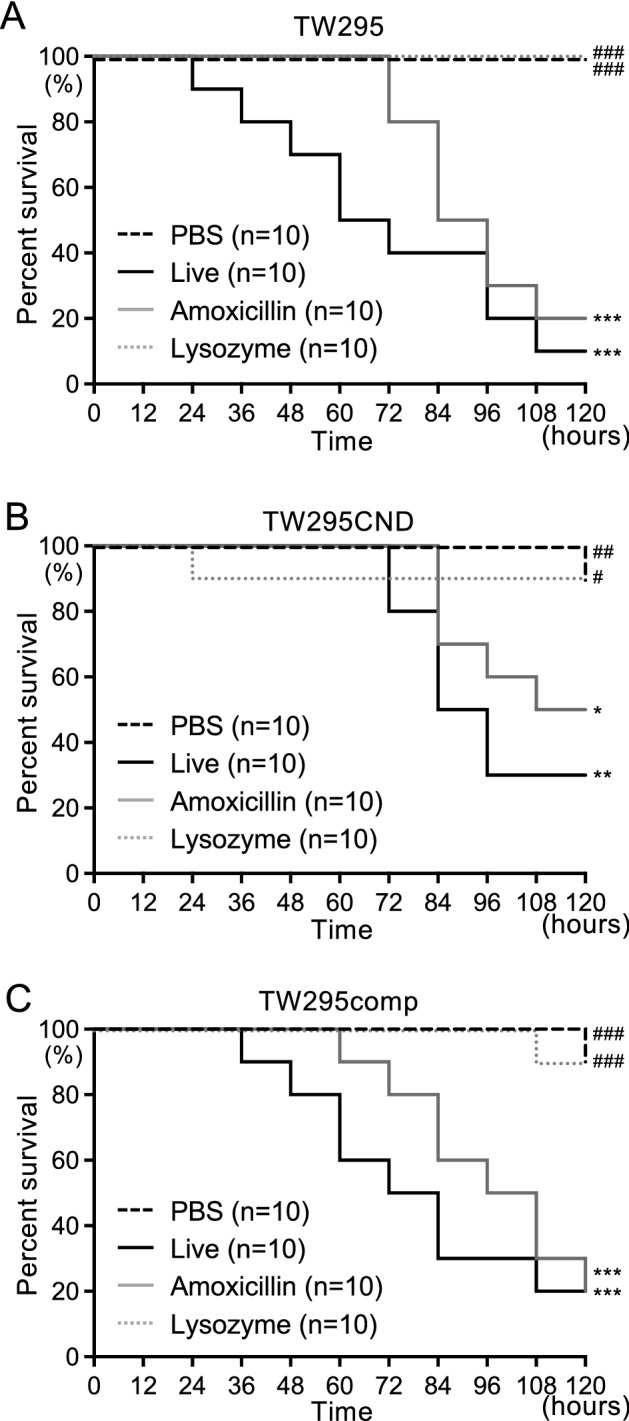
Survival curve for silkworm larvae administered with live *S. mutans*, amoxicillin-killed *S. mutans*, and lysozyme-killed *S. mutans*. Silkworm larvae infected with 1 × 10^7^ CFU of (**A**) TW295 (Cnm +), (**B**) TW295CND (Cnm−), and (**C**) TW295comp (Cnm +). Survival rates in each group were evaluated in a Kaplan–Meier plot, which was analyzed by a log-rank test. ^*^P < 0.05, ^**^P < 0.01 and ^***^P < 0.001 versus phosphate-buffered saline group; ^#^*P* < 0.05, ^##^*P* < 0.01, and ^###^*P* < 0.001 versus live *S. mutans* administration group.

Live *S. mutans* and killed *S. mutans* treated with amoxicillin or lysozyme were all adjusted to 1 × 10^7^ CFU and administered to silkworms. The silkworms administered with amoxicillin-killed TW295 showed delayed mortality compared with those administered with live bacteria (Fig. 4A). However, the survival rate of silkworms administered with amoxicillin-treated TW295 was significantly lower than that of silkworms administered with phosphate buffered saline (PBS) (*P* < 0.001), and 50% of the silkworms died 84 h after the start of the experiment. In contrast, administration of lysozyme-killed TW295 did not reduce the survival rate of the silkworms. TW295CND administration also resulted in a significant decrease in survival rate in the groups treated with live and amoxicillin-killed bacteria compared with the control group (*P* < 0.05) (Fig. 4B). However, the survival rate of silkworms treated with TW295CND was higher than that of silkworms treated with TW295 for both live and amoxicillin-killed bacteria. Administration of lysozyme-killed TW295CND did not reduce the survival rate of silkworms. Furthermore, TW295comp administration restored the virulence of the silkworms in both live and amoxicillin-killed bacteria compared with those administered with TW295CND (Fig. 4C).

### Histopathological evaluation of silkworms administered with live *S. mutans* and killed *S. mutans* (treated with amoxicillin or lysozyme)

**Figure 5:**
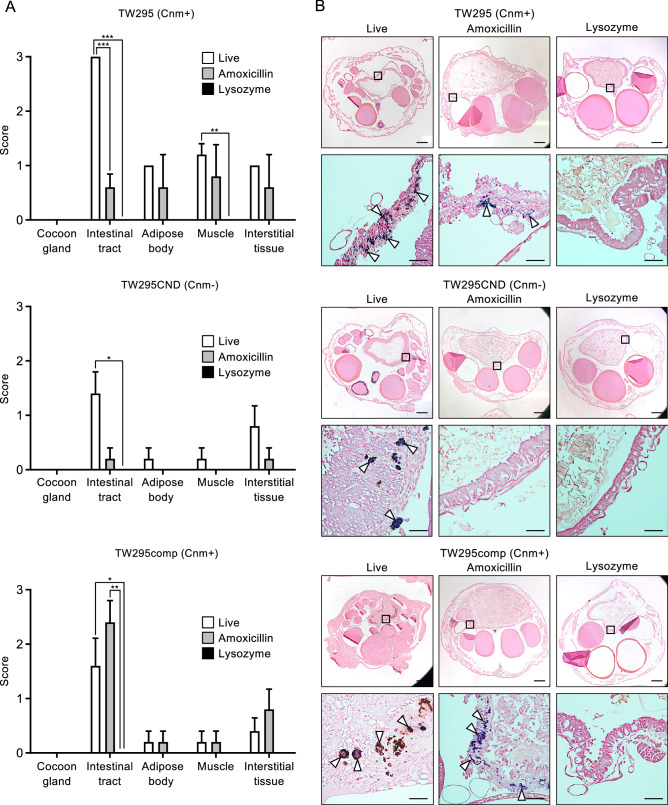
Histopathological evaluation of silkworm larvae administered with live *S. mutans*, amoxicillin-killed *S. mutans*, and lysozyme-killed *S. mutans*. (**A**) Scoring for the localization of bacteria in tissue sections of each organ. Significant differences were observed using analysis of variance with Bonferroni correction (*P < 0.05, **P < 0.01, and ***P < 0.001). (**B**) Representative histopathological images following Gram staining of tissue sections of the intestinal tract from silkworms euthanized 72 h after *S. mutans* administration. Lower panels show high-magnification images of the boxes on the upper images. White arrowheads indicate bacterial masses. Bar = 1 mm (upper images) and bar = 100 μm (lower images).

To confirm the presence of *S. mutans* in silkworm tissues after administration of the bacteria, histopathological evaluation was performed by preparing tissue sections from euthanized silkworms. In silkworms administered with live TW295, bacteria were found in each organ except the cocoon gland, and the highest numbers of bacteria were found in the intestinal tract (Fig. 5A,B). Silkworms administered with amoxicillin-killed TW295 had fewer bacteria than silkworms administered with live TW295, but bacteria were confirmed in all organs. In contrast, no bacteria were found in any of the organs of silkworms administered with lysozyme-killed TW295. In silkworms treated with live TW295CND, bacteria were found in all organs except the cocoon gland. However, silkworms treated with amoxicillin-killed TW295CND showed only a few bacteria in the intestinal tract and interstitial tissue, and those treated with lysozyme-killed TW295CND had no bacteria in any organ. Furthermore, bacteria were detected in all organs except the cocoon gland in silkworms treated with live and amoxicillin-killed TW295comp, whereas no bacteria were detected in any organ when lysozyme-killed bacteria were administered. Histopathological evaluation of each organ of the silkworms, except for bacterial localization, is shown in Supplementary Tables 1–3.

### Collagen-binding activity and silkworm larvae virulence assay of live and killed *S. mutans* clinical isolates

**Figure 6:**
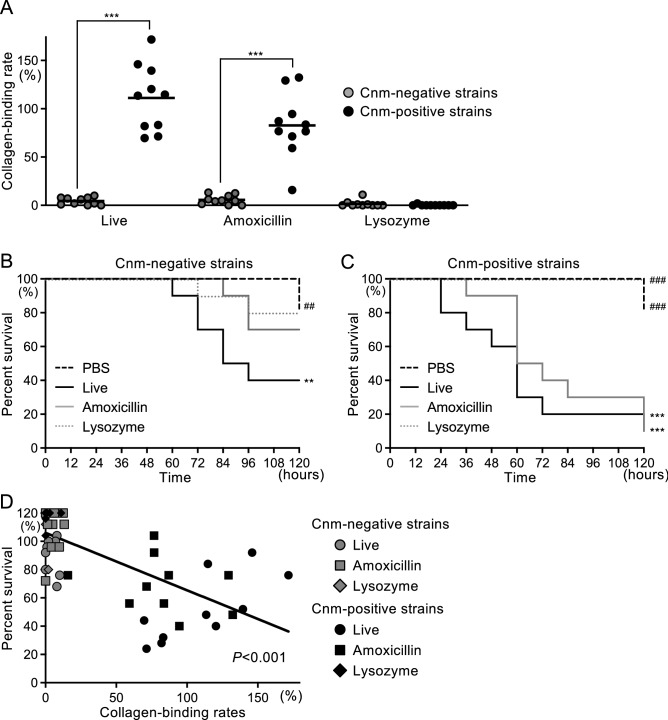
Collagen-binding properties and survival curve for silkworm larvae induced by live *S. mutans* clinical isolates and killed *S. mutans* clinical isolates (treated with amoxicillin or lysozyme). (**A**) Collagen-binding rates of *S. mutans* strains. Each closed circle represents the mean value for each bacterial strain. Significant differences were observed using analysis of variance with Bonferroni correction (***P < 0.001). Silkworm larvae infected with (**B**) Cnm-negative clinical isolates, and (**C**) Cnm-positive clinical isolates. Survival rates in each group were evaluated in a Kaplan–Meier plot, which was analyzed by a log-rank test. ^**^P < 0.01 and ^***^P < 0.001 versus phosphate-buffered saline group; ^##^*P* < 0.01 and ^###^*P* < 0.001 versus live *S. mutans* administration group. (**D**) Correlation between collagen-binding rates and survival rates of silkworms analyzed by regression analysis. Each point represents the mean value for each bacterial strain.

The collagen-binding activity of 10 Cnm-positive and 10 Cnm-negative *S. mutans* clinical isolates was evaluated. The Cnm-negative group had almost no collagen-binding activity in live *S. mutans*, amoxicillin-killed *S. mutans*, or lysozyme-killed *S. mutans* (Fig. 6A). In contrast, high collagen-binding rates were observed in live bacteria in the Cnm-positive group, whose collagen-binding rate was significantly higher than that of the Cnm-negative group (*P* < 0.001). Collagen-binding activity was also observed in the amoxicillin-killed Cnm-positive group, but not in the lysozyme-killed group.

The live bacteria in the Cnm-negative group killed half of the silkworms in 84 h, but more than 70% and 80% of the amoxicillin-killed and lysozyme-killed silkworms survived throughout the experiment, respectively (Fig. 6B). The live and amoxicillin-killed bacteria in the Cnm-positive group killed more than half of the silkworms in 60 h (Fig. 6C). However, the lysozyme-killed bacteria did not show any decrease in survival rate compared with the control. In these clinical strains, there was a correlation between collagen-binding activity and reduced survival rate of the silkworms (*P* < 0.001) (Fig. 6D).

## Discussion

*S. mutans* is involved in the development of cardiovascular diseases such as IE and intracerebral hemorrhage, when they infiltrate into the bloodstream from the oral cavity^8,17^. Although bacterial adherence of *S. mutans* to cardiac tissue is important for the development of cardiovascular disease^18^, it remains unknown whether dead *S. mutans* have these adhesive properties. In the present study, we found that the collagen-binding ability and the adhesion and virulence to silkworms were present in bacteria killed by amoxicillin treatment, especially Cnm-positive *S. mutans*. In contrast, lysozyme treatment was found to be effective in eliminating the virulence of all *S. mutans* strains.

First, we killed *S. mutans* strains with amoxicillin, which is a major antibiotic widely used to prevent IE during invasive dental treatment^4^. Electron microscopic images revealed abnormal findings on the cell surface and cytoplasm of *S. mutans* killed with amoxicillin. However, we found that amoxicillin-treated Cnm-positive *S. mutans* retained its collagen-binding ability and virulence to silkworms. These results suggest that preoperative administration of amoxicillin during invasive dental treatment reduces, but does not eliminate, the pathogenicity of *S. mutans* against cardiovascular diseases. Based on the results obtained from our study, the effects of other antibiotics on the pathogenicity of *S. mutans* should be analyzed.

Lysozyme is known to be a major antimicrobial agent in saliva and serum, and lysozyme has also been applied in food and pharmaceuticals^2,19^. Cnm-positive *S. mutans* lysed with lysozyme lost its collagen-binding ability and virulence against silkworms, and the inhibitory effect of lysozyme on *S. mutans* was greater than that of amoxicillin. Therefore, lysozyme may be more effective than antibiotics in inhibiting cardiovascular diseases caused by Cnm-positive *S. mutans*. In addition to lysozyme, there are other antimicrobial substances such as lactoferrin and lactoperoxidase in saliva and serum^20–22^ that are effective in inhibiting the cariogenicity of *S. mutans*^23^. Therefore, the inhibitory effect of these antimicrobial substances derived from humans on the pathogenicity of *S. mutans* in cardiovascular diseases should be analyzed.

In some patients with cardiovascular diseases including IE, the bacterial DNA of *S. mutans* has been detected in heart valve specimens, even though live *S. mutans* was not isolated by blood culture methods^14^. However, it was unclear whether the detection of dead *S. mutans* DNA meant that *S. mutans* was attached to the tissue surface via collagen or that there were dead nonpathogenic *S. mutans* that were captured and lysed by immune cells. In the present study, we evaluated the adhesion of *S. mutans* to silkworm organs histopathologically. The results showed that Cnm-positive *S. mutans* killed with amoxicillin had the ability to adhere to silkworm organs, while lysozyme-treated bacteria were not found in silkworm organs. This suggests that the pathogenicity of dead *S. mutans* detected in cardiovascular disease lesions may vary depending on how they were killed. However, it is known that silkworm hemoproteins can bind to various bacteria and form nodules^24^. Therefore, it is necessary to use silkworms to analyze in detail whether the ability of killed *S. mutans* to adhere to tissues is due to the collagen-binding ability or to the cellular immune response.

*Staphylococcus aureus* and *Enterococcus faecalis* are the major causative agents of IE^4^. *S. aureus* has adhesion properties to osteoblasts and kidney cells, even when the bacteria are killed by ultraviolet rays or formalin^25,26^. Additionally, *E. faecalis* killed by antibiotic treatment was able to adhere to the artificially injured heart valves of rats, resulting in the development of IE^27^. These bacteria express collagen-binding proteins homologous to Cnm on the cell surface^10^, and may be involved in bacterial adhesions. Although one study focused on the presence of fibronectin-binding activity in killed *S. aureus* treated with formalin^25^, no study to date has focused on the collagen-binding activity of killed bacteria. Thus, to the best of our knowledge, this is the first study to clearly show that the collagen-binding protein of killed bacteria is involved in bacterial adhesion.

The strength of the collagen-binding ability of Cnm-positive *S. mutans* varied among strains^10^, and this was also observed in killed bacteria. The strength of the collagen-binding ability is related to the strength of the expression of mRNA encoding Cnm^9^. Additionally, the collagen-binding ability of Cnm-positive *S. mutans* is affected by PA, a 190 kDa cell surface protein antigen^11^. Therefore, it is necessary to focus on the proteins that affect the expression of Cnm such as PA as well as Cnm, and to analyze the expression pattern of Cnm in each *S. mutans* strain in detail, not only in live bacteria but also in dead bacteria.

*S. mutans* of different genotypes are present in the oral cavity^28^, and there may be some participants in whom both Cnm-positive and Cnm-negative *S. mutans* are present in the oral cavity, but the details remain unknown. There are some reports of increased aggregation ability as a result of co-cultivation of several different bacterial species^29,30^. Cnm-positive-*S. mutans* may bind to collagen and serve as a scaffold for other Cnm-negative *S. mutans*, or an aggregation reaction may occur between dead and live bacteria. In future research, it will be necessary to clarify how often Cnm-positive *S. mutans* and Cnm-negative *S. mutans* are mixed, and in what proportion each bacterium exists in the oral cavity. Additionally, collagen-binding assays and silkworm models using multiple *S. mutans* strains should be evaluated.

Recently, it has been shown that Cnm-positive *S. mutans* in the oral cavity affect not only cardiovascular diseases, but also inflammatory bowel disease, non-alcoholic steatohepatitis, and IgA nephropathy^31–33^. In these systemic diseases, Cnm-positive *S. mutans* in the oral cavity infiltrating into the bloodstream is considered to be a common trigger for the onset of the disease. According to the results of the present study, dead Cnm-positive *S. mutans* in the oral cavity may be a risk factor for the development of systemic diseases via the blood vessels, although the pathogenicity may be lower than that for live bacteria.

In summary, Cnm-positive *S. mutans* killed with amoxicillin has collagen-binding ability and virulence in silkworms, although these pathogenicities are lost in the bacteria killed with lysozyme. These results suggest that antimicrobial substances derived from humans may be more effective than antibiotics in preventing the development of cardiovascular diseases caused by Cnm-positive *S. mutans*.

## Methods

### Ethics statement

This study was conducted in full adherence to the Declaration of Helsinki. The study protocol was approved by the Ethics Committee of Osaka University Graduate School of Dentistry (approval no. 04382). All *S. mutans* strains have been used in our previous studies, and informed consent was obtained from participants (and parents, if necessary) at the time of oral specimen collection, which could be referred in the previous manuscripts^28,34–38^.

### Bacterial strains and growth conditions

*S. mutans* strains used in the present study are listed in Supplementary Table 4. *S. mutans* TW295 (Cnm +) isolated from a patient with bacteremia after tooth extraction were used^28^. TW295CND, a Cnm-knockout mutant strain of TW295, and TW295comp, a Cnm-complemented strain of TW295, were also used, and were generated as previously described^37,39^. Furthermore, a total of 20 *S. mutans* clinical strains selected from our laboratory stock (10 each displaying Cnm-positive and Cnm-negative phenotypes) were used. All strains were confirmed to be *S. mutans* based on observation of rough colony morphology on Mitis-salivarius agar plates (Difco Laboratories, Detroit, MI, USA) containing bacitracin (0.2 U/ml; Sigma-Aldrich Co., St. Louis, MO, USA) and 15% (wt/vol) sucrose (MSB agar) as well as 16S rRNA sequence analysis with the primers 8UA (5′-AGA GTT TGA TCC TGG CTC AG-3′) and 1540R (5′-AAG GAG GTG ATC CAG CC-3′), as described previously^40^. For routine growth, all strains were cultured overnight in brain heart infusion broth (Difco Laboratories). When the mutant strains of TW295 were cultured, TW295CND was supplemented with erythromycin (10 µg/ml), and TW295comp was supplemented with erythromycin (10 µg/ml) and spectinomycin (10 mg/ml).

### Preparations of killed bacteria

Cultured bacteria were collected by centrifugation at 3,000 × rpm at 4 °C for 10 min. For the lysozyme and amoxicillin treatment, the cultures were washed and resuspended in PBS containing 10 mg/mL lysozyme (FUJIFILM Wako Pure Chemical Corporation, Tokyo, Japan) and 1.0 mg/mL amoxicillin (FUJIFILM Wako Pure Chemical Corporation), respectively, and incubated at 37 °C for 18 h. Successful killing of the bacteria was confirmed by the absence of colonies on MSB agar after culturing at 37 °C for 48 h. After the lysozyme and amoxicillin treatment, each bacterial suspension was washed twice and resuspended in PBS to reach an optical density at 550 nm (OD_550_) of 1.0, which was equal to 1 × 10^9^ CFU/ml^41^. The bacterial suspension was diluted and used in the following study.

### Electron microscopy observations

Observation using electron microscopy was performed in accordance with the method previously described^34,42^. As preparation for SEM imaging, each bacterial sample was washed and fixed with 2% osmium tetroxide and 1% glutaraldehyde, dehydrated with ethanol, and then dried with *t*-butyl alcohol by the freeze-drying method. The dried samples were mounted on the stage and coated with osmium for conductive processing and then observed with SEM. In the TEM analysis, each bacterial sample was washed and fixed with 2% glutaraldehyde adjusted with PBS. After dehydration, bacterial cells were embedded in Epon, then cut into ultrathin sections, and the bacterial structure of these samples was observed by TEM.

### Collagen-binding assay

The collagen-binding properties of the *S. mutans* strains were evaluated according to methods described previously, with some modifications^9^. A 10 mg/ml sample of type I collagen (Sigma-Aldrich Co.) prepared in 0.25 M acetic acid was coated onto 96-well tissue culture plates (Becton Dickinson, Franklin Lakes, NJ, USA) and incubated overnight at 4 °C. The plates were then washed three times with PBS and blocked for 1.5 h with bovine serum albumin (BSA; Sigma-Aldrich Co.) in PBS at 37 °C. Cultured bacteria was collected by centrifugation and washed and diluted with PBS. Then, 100 µL of the bacterial suspension was added to the wells (1 × 10^9^ CFU per well). After 3 h of incubation at 37 °C, adherent cells were washed three times with PBS and fixed with 100 µL of 25% formaldehyde at room temperature for 30 min. After another three washes with PBS, adherent cells were stained with 100 µL of 0.05% crystal violet (FUJIFILM Wako Pure Chemical Corporation, Tokyo, Japan) in water for 1 min, washed three times with PBS, and the dye was dissolved by adding 7% acetic acid (100 µL) before determining the OD_595_ values. Results are expressed as OD_595_ values following subtraction of those from BSA-coated wells. The results for each strain are expressed as a percentage compared with the binding property of SA83 (Cnm +), which was defined as 100%. Data are expressed as the mean ± standard deviation of four independent experiments using three wells for each sample.

### Fluorescence microscopy observations

Observation of *S. mutans* strains binding to type I collagen using confocal laser scanning microscopy was performed by a method described previously^43^, with some modifications. The collagen-binding assay described above was performed using a chambered cover glass system (CultureWell™, Grace Bio Labs, Bend, OR, USA) instead of a 96-well plate. After binding the bacteria to collagen for 3 h, bacterial cells were stained with 5 µl of 10 mM hexidium iodide (Invitrogen, Carlsbad, CA, USA) in 1 ml of Hanks’ balanced salt solution (Lonza, Walkersville, MD, USA) for 15 min at room temperature in the dark. Stained bacteria were observed by confocal scanning laser microscopy using a TCS-SP5 microscope (Leica Microsystems GmbH, Wetzlar, Germany) with reflected laser light at 543 nm, as well as a DMI6000 B fluorescence microscope (Leica Microsystems GmbH) and a 63 × oil immersion objective.

### Silkworm larvae virulence assay

A silkworm larvae virulence assay was performed by a method described previously^34^, with some modifications. *B. mori* larvae aged 10 days were purchased (Kougensya, Nagano, Japan), and then stored at 25 °C in the dark. At 18 days of age, larvae with body weights in the range of 150–250 mg were randomly divided into each group (10 larvae per group). The larvae were inoculated on the dorsal surface with 50 µL of bacterial suspension containing different amounts of live *S. mutans* (1 × 10^5^ CFU, 1 × 10^6^ CFU, and 1 × 10^7^ CFU, respectively). After injection, the silkworms were incubated at 37 °C for 120 h. Live *S. mutans* and killed *S. mutans* treated with amoxicillin or lysozyme, adjusted to 1 × 10^7^ CFU, were also administered to silkworms in the same manner. The larvae were checked every 12 h and were considered dead if they did not move in response to touch. In addition, 20 *S. mutans* clinical isolates were inoculated into n = 3 silkworms each and the average time of death of the three silkworms was calculated. All the experiments were performed three times in each group to ensure reproducibility.

### Histopathological evaluation of silkworm larvae

Silkworms euthanized 72 h after *S. mutans* administration were fixed in 10% formalin neutral buffer solution (FUJIFILM Wako Pure Chemical Corporation) and divided into five sections from head to tail at intervals of 7 mm. These specimens were embedded in paraffin, and cut into 3 μm sections. Gram staining and hematoxylin–eosin staining were then performed using these sections, followed by evaluation of histopathological features as shown in Supplementary Tables 1–3.

For scoring of bacterial infection and pigmentation, the entire specimens of five sections of the silkworm were observed, and the area with the highest bacterial accumulation or pigmentation in each organ was extracted. Then, the area was observed under a 10 × field of view of an objective lens, and the number of sites with bacterial mass or pigmentation was counted to obtain the following scores: score 0 (no bacterial mass or pigmentation), score 1 (5 or less), score 2 (6 to 10), score 3 (11 or more). The scoring of degenerative lesions was based on the number of sections with lesions among the five sections, as follows: score 0 (no lesions), score 1 (1 section), score 2 (2 to 3 sections), score 3 (4 to 5 sections). Necrosis was scored based on the number of sections with lesions among the five cross-sections as follows: score 0 (no lesions), score 1 (1 section), score 2 (2 sections), score 3 (3 to 5 sections). All scoring evaluations were performed in a double-blinded fashion by a pathologist (Sept. Sapie Co. Ltd, Tokyo, Japan).

### Statistical analysis

Statistical analyses were performed using GraphPad Prism 9 (GraphPad Software Inc., La Jolla, CA, USA) by a method described previously^44^, with some modifications. Intergroup differences were compared using analysis of variance (ANOVA). Bonferroni correction was used for post hoc analyses. Differences with *P* < 0.05 were considered statistically significant. Survival rates in the silkworm larvae virulence assay in each group were evaluated with a Kaplan–Meier plot, which was analyzed using a log-rank test.

## Supplementary Information

Supplementary Information.
