# Supplementary material for: Evaluation of the collagen-binding properties and virulence of killed Streptococcus mutans in a silkworm model
Source: Sci Rep. 2022 Feb 18;12:2800. doi: 10.1038/s41598-022-06345-x (PMC8857238; doi:10.1038/s41598-022-06345-x)
Supplement: Supplementary file 1 — Supplementary Information. [file 41598_2022_6345_MOESM1_ESM.pdf]

# Evaluation of the collagen-binding properties and virulence of killed *Streptococcus mutans* in a silkworm model

Yuto Suehiro, Ryota Nomura, Saaya Matayoshi, Masatoshi Otsugu, Naoki Iwashita & Kazuhiko Nakano

**Supplementary Table 1** Histopathological evaluations of transversal sections of silkworms administered live *S. mutans* TW295, and the killed bacteria treated by lysozyme or amoxicillin.

| Finding | Live (n=5) | Amoxicillin (n=5) | Lysozyme (n=5) | PBS (n=5) |
|---|---|---|---|---|
| Cocoon gland | | | | |
| Edema-like degeneration | 0.00 ± 0.00 | 0.40 ± 0.40 | 0.00 ± 0.00 | 0.40 ± 0.24 |
| Pigmentation | 0.00 ± 0.00 | 0.00 ± 0.00 | 0.00 ± 0.00 | 0.00 ± 0.00 |
| Epithelial necrosis | 0.00 ± 0.00 | 0.20 ± 0.00 | 0.00 ± 0.00 | 0.00 ± 0.00 |
| Intestinal tract | | | | |
| Pigmentation | 0.00 ± 0.00 | 0.80 ± 0.37 | 0.20 ± 0.20 | 0.20 ± 0.20 |
| Degeneration (clear cell), basal cell | 0.00 ± 0.00 | 0.00 ± 0.00 | 0.00 ± 0.00 | 0.00 ± 0.00 |
| Mucosal epithelial detachment | 1.40 ± 0.24** | 0.60 ± 0.60 | 0.00 ± 0.00## | 0.00 ± 0.00## |
| Mucosal epithelial necrosis | 0.00 ± 0.00 | 0.60 ± 0.00 | 0.00 ± 0.00 | 0.60 ± 0.00 |
| Adipose body | | | | |
| Pigmentation | 0.20 ± 0.20 | 0.00 ± 0.00 | 0.00 ± 0.00 | 0.00 ± 0.00 |
| Degeneration, vitreous drop | 0.00 ± 0.00 | 0.00 ± 0.00 | 0.00 ± 0.00 | 0.00 ± 0.00 |
| Edema-like degeneration | 0.00 ± 0.00 | 0.20 ± 0.20 | 0.00 ± 0.00 | 0.00 ± 0.00 |
| Atrophy | 0.00 ± 0.00 | 0.00 ± 0.00 | 0.00 ± 0.00 | 0.00 ± 0.00 |
| Muscle | | | | |
| Pigmentation | 0.00 ± 0.00 | 0.20 ± 0.20 | 0.20 ± 0.20 | 0.00 ± 0.00 |
| Interstitial tissue | | | | |
| Pigmentation | 1.00 ± 0.00* | 0.40 ± 0.24 | 0.00 ± 0.00# | 0.20 ± 0.20# |

Data are expressed as the mean ± SE.
Supplementary Table 1 to 3 are analyses conducted simultaneously, and PBS data is duplicated in each Table.
Significant differences were observed using analysis of variance with Bonferroni correction. *$P < 0.05$ and **$P < 0.01$ versus phosphate-buffered saline group; #$P < 0.05$ and ##$P < 0.01$ versus live *S. mutans* administration group.

**Supplementary Table 2** Histopathological evaluations of transversal sections of silkworms administered live *S. mutans* TW295CND, and the killed bacteria treated by lysozyme or amoxicillin.

| Finding | Live (n=5) | Amoxicillin (n=5) | Lysozyme (n=5) | PBS (n=5) |
|---|---|---|---|---|
| Cocoon gland | | | | |
| Edema-like degeneration | 0.80 ± 0.37 | 0.40 ± 0.40 | 0.20 ± 0.20 | 0.40 ± 0.24 |
| Pigmentation | 0.00 ± 0.00 | 0.00 ± 0.00 | 0.00 ± 0.00 | 0.00 ± 0.00 |
| Epithelial necrosis | 0.00 ± 0.00 | 0.00 ± 0.00 | 0.00 ± 0.00 | 0.00 ± 0.00 |
| Intestinal tract | | | | |
| Pigmentation | 1.20 ± 0.37 | 0.20 ± 0.20 | 0.00 ± 0.00# | 0.20 ± 0.20 |
| Degeneration (clear cell), basal cell | 0.20 ± 0.00 | 0.00 ± 0.00 | 0.00 ± 0.00 | 0.00 ± 0.00 |
| Mucosal epithelial detachment | 0.80 ± 0.58 | 0.00 ± 0.00 | 0.00 ± 0.00 | 0.00 ± 0.00 |
| Mucosal epithelial necrosis | 0.60 ± 0.00 | 0.00 ± 0.00 | 0.20 ± 0.00 | 0.60 ± 0.00 |
| Adipose body | | | | |
| Pigmentation | 0.20 ± 0.20 | 0.00 ± 0.00 | 0.00 ± 0.00 | 0.00 ± 0.00 |
| Degeneration, vitreous drop | 0.00 ± 0.00 | 0.00 ± 0.00 | 0.00 ± 0.00 | 0.00 ± 0.00 |
| Edema-like degeneration | 0.00 ± 0.00 | 0.00 ± 0.00 | 0.00 ± 0.00 | 0.00 ± 0.00 |
| Atrophy | 0.00 ± 0.00 | 0.00 ± 0.00 | 0.00 ± 0.00 | 0.00 ± 0.00 |
| Muscle | | | | |
| Pigmentation | 0.20 ± 0.20 | 0.00 ± 0.00 | 0.00 ± 0.00 | 0.00 ± 0.00 |
| Interstitial tissue | | | | |
| Pigmentation | 0.40 ± 0.24 | 0.40 ± 0.24 | 0.80 ± 0.20 | 0.20 ± 0.20 |

Data are expressed as the mean ± SE.

Supplementary Table 1 to 3 are analyses conducted simultaneously, and PBS data is duplicated in each Table.

Significant differences were observed using analysis of variance with Bonferroni correction. $^{\#}P < 0.05$ versus live *S. mutans* administration group.

**Supplementary Table 3** Histopathological evaluations of transversal sections of silkworms administered live *S. mutans* TW295comp, and the killed bacteria treated by lysozyme or amoxicillin.

| Finding | Live (n=5) | Amoxicillin (n=5) | Lysozyme (n=5) | PBS (n=5) |
|---|---|---|---|---|
| Cocoon gland | | | | |
| Edema-like degeneration | 1.00 ± 0.55 | 1.20 ± 0.20 | 0.00 ± 0.00 | 0.40 ± 0.24 |
| Pigmentation | 0.20 ± 0.00 | 0.00 ± 0.00 | 0.00 ± 0.00 | 0.00 ± 0.00 |
| Epithelial necrosis | 0.20 ± 0.00 | 0.40 ± 0.00 | 0.00 ± 0.00 | 0.00 ± 0.00 |
| Intestinal tract | | | | |
| Pigmentation | 1.40 ± 0.68 | 2.00 ± 0.45 | 0.20 ± 0.20 | 0.20 ± 0.20 |
| Degeneration (clear cell), basal cell | 1.20 ± 0.00 | 0.00 ± 0.00 | 0.00 ± 0.00 | 0.00 ± 0.00 |
| Mucosal epithelial detachment | 0.80 ± 0.58 | 1.00 ± 0.63 | 0.00 ± 0.00 | 0.00 ± 0.00 |
| Mucosal epithelial necrosis | 0.00 ± 0.00 | 1.40 ± 0.00 | 0.60 ± 0.00 | 0.60 ± 0.00 |
| Adipose body | | | | |
| Pigmentation | 0.20 ± 0.20 | 0.20 ± 0.20 | 0.00 ± 0.00 | 0.00 ± 0.00 |
| Degeneration, vitreous drop | 0.40 ± 0.00 | 0.00 ± 0.00 | 0.00 ± 0.00 | 0.00 ± 0.00 |
| Edema-like degeneration | 0.00 ± 0.00 | 0.00 ± 0.00 | 0.00 ± 0.00 | 0.00 ± 0.00 |
| Atrophy | 0.00 ± 0.00 | 0.00 ± 0.00 | 0.00 ± 0.00 | 0.00 ± 0.00 |
| Muscle | | | | |
| Pigmentation | 0.20 ± 0.20 | 0.40 ± 0.40 | 0.00 ± 0.00 | 0.00 ± 0.00 |
| Interstitial tissue | | | | |
| Pigmentation | 0.40 ± 0.24 | 0.80 ± 0.20 | 0.40 ± 0.24 | 0.20 ± 0.20 |

Data are expressed as the mean ± SE.

Supplementary Table 1 to 3 are analyses conducted simultaneously, and PBS data is duplicated in each Table.

**Supplementary Table 4** *S. mutans* strains used in the present study.

| Strain | Serotype | Features | Isolated countries | Cnm | Reference |
|---|---|---|---|---|---|
| TW295 | *k* | Blood isolate from a patient with bacteremia after tooth extraction | Japan | + | 35 |
| TW295CND | *k* | $Em^r$; Cnm-knockout strain of TW295 | Japan | - | 28 |
| TW295comp | *k* | $Em^r$, $Spe^r$; Cnm-complemented strain of TW295 | Japan | + | 28 |
| NN2101 | *c* | Oral isolate from a healthy subject | Japan | - | 28 |
| NN2004 | *c* | Oral isolate from a healthy subject | Japan | - | 28 |
| NN2006 | *c* | Oral isolate from a healthy subject | Japan | - | 28 |
| NN2007 | *c* | Oral isolate from a healthy subject | Japan | - | 28 |
| NN2009 | *c* | Oral isolate from a healthy subject | Japan | - | 28 |
| NN2010 | *c* | Oral isolate from a healthy subject | Japan | - | 28 |
| NN2012 | *c* | Oral isolate from a healthy subject | Japan | - | 28 |
| SA23 | *c* | Oral isolate from a healthy subject | Finland | - | 34 |
| TLJ5-1 | *c* | Oral isolate from a healthy subject | Thailand | - | 34 |
| TLJ6-1 | *c* | Oral isolate from a healthy subject | Thailand | - | 36 |
| NN2095 | *c* | Oral isolate from a healthy subject | Japan | + | 28 |
| NN2115 | *e* | Oral isolate from a healthy subject | Japan | + | 28 |
| NN2103 | *f* | Oral isolate from a healthy subject | Japan | + | 28 |
| NN1005 | *c* | Oral isolate from a healthy subject | Japan | + | 28 |
| NN2141 | *f* | Oral isolate from a healthy subject | Japan | + | 28 |
| LJ24 | *f* | Oral isolate from a healthy subject | Japan | + | 38 |
| LJ32 | *f* | Oral isolate from a healthy subject | Japan | + | 38 |
| SA63 | *e* | Oral isolate from a healthy subject | Finland | + | 37 |
| TLJ34-1 | *f* | Oral isolate from a healthy subject | Thailand | + | 36 |
| TLJ72-3 | *k* | Oral isolate from a healthy subject | Thailand | + | 36 |
| SA83 | *c* | Oral isolate from a healthy subject | Finland | + | 37 |
